# Effect of biologic materials on the outcomes of horizontal alveolar ridge augmentation: A retrospective study

**DOI:** 10.1002/cre2.343

**Published:** 2020-11-04

**Authors:** Janina Golob Deeb, Amy Reichert, Caroline K. Carrico, Daniel M. Laskin, George R. Deeb

**Affiliations:** ^1^ Department of Periodontics School of Dentistry, Virginia Commonwealth University Richmond Virginia USA; ^2^ Cardinal Dental Specialists Harrisonburg Virginia USA; ^3^ Dental Public Health and Policy, Oral Health Research Core Virginia Commonwealth University Richmond Virginia USA; ^4^ Department of Oral and Maxillofacial Surgery School of Dentistry, Virginia Commonwealth University Richmond Virginia USA

**Keywords:** biologics, bone density, bone gain, horizontal ridge augmentation, PRP, radiographic evaluation, rhPDGF‐BB

## Abstract

**Purpose:**

The purpose of this study was to investigate if the addition of biologic agents to a particulate bone graft enhances horizontal ridge augmentation outcomes in terms of bone dimensions, bone density, and successful implant placement.

**Materials and Methods:**

A retrospective chart review was done to assess the clinical and radiographic outcomes in 52 horizontal ridge augmentation sites in 43 patients. Information was gathered regarding surgical technique, type of graft material, biologic agents used (PRP or rhPDGF‐BB), method of space maintenance, and achieved alveolar ridge width and bone density changes as quantified on CBCT scans.

**Results:**

The use of tenting screws, a resorbable membrane, and a combination of particulate allogenic and xenogenic bone graft material provided an average horizontal bone gain of 3.6 mm in the 52 augmented sites. There was no statistically significant difference observed in the amount of horizontal bone gain between sites treated with the addition of biologic agents (*n* = 21), or with a particulate bone graft alone (*n* = 31). A marginally statistically significant difference was found in the density of the grafted bone with the addition of biologics (*p* value = .0653).

**Conclusion:**

The addition of biologic agents to the graft materials did not have a significant effect on the amount of horizontal bone gain or successful implant placement; however, it marginally enhanced the bone density of the grafted area.

## 1 INTRODUCTION

Following tooth extraction, resorption of the alveolar ridge in a vertical and horizontal dimension occurs almost immediately in the majority of patients (Schropp, Wenzel, Kostopoulos, & Karring, 2003; Van Der Weijden, Dell'Acqua, & Slot, 2009), resulting in alveolar ridge width and height loss (Lekovic et al., 1998; Lindhe, 2005). Severely atrophic sites generally do not provide adequate bone support for placement of a dental implant of appropriate size in an optimal position and may require a bone augmentation procedure depending on the defect type(Benic & Hämmerle, 2014). To resolve this problem, techniques of horizontal augmentation of the alveolar ridge have been developed (Aghaloo & Moy, 2007; Beitlitum, Artzi, & Nemcovsky, 2010; Esposito et al., 2009; McAllister & Haghighat, 2007). Bone autografts, allografts, and xenografts are used to serve as a scaffold for angiogenesis and osteogenesis, while tenting screws and reinforced barriers provide significant rigid space maintenance and help define the area of augmentation for implant placement (Le & Rohrer, 2017; Wang & Boyapati, 2006). Guided bone regeneration, with particulate graft materials and resorbable collagen membranes, is also an effective technique for horizontal alveolar ridge augmentation (Wessing, Lettner, & Zechner, 2018). With the intent of improving new bone formation, biologic agents are often added to the bone graft (Badylak et al., 2017; Lekovic et al., 2003; Del Amo, Monje, Padial‐Molina, Tang, & Wang, 2015; Eskan et al., 2014; Yoon, Lee, & Yoon, 2014). These agents select for certain types of cells needed for faster and more efficient regeneration of the alveolar bone and potentially enhance the regeneration process. The most commonly used biologics in periodontal and alveolar bone regeneration include platelet‐rich plasma (PRP) and recombinant human platelet‐derived growth factor‐ββ (rhPDGF‐BB).(2017; Eskan et al., 2014; Eskan & Greenwell, 2011; Lekovic et al., 2012; Papadopoulos, Dereka, Vavouraki, & Vrotsos, 2003; Sarment et al., 2006).

Platelet‐rich plasma (Harvest Terumo BCT, Lakewood, CO) is derived from autologous blood and contains numerous biologically active growth factors, including PDGF‐AB, PDGF‐BB, transforming growth factor‐β (TGF‐B), vascular endothelial growth factor (VEG‐F), connective tissue growth factor (CTGF), insulin‐like growth factor (IGF), and endothelial growth factor (EGF), which are thought to enhance soft tissue healing, bone augmentation, and periodontal regeneration (Albanese, Licata, Polizzi, & Campisi, 2013; Nevins et al., 2005). PRP has demonstrated enhanced bone regeneration and increased horizontal bone gain and percentage of vital bone in a clinical trial (Eskan et al., 2014).

Recombinant human PDGF‐BB is a highly purified synthetic bioactive protein that has been credited with promoting chemotaxis, mitogenesis, and angiogenesis (Hollinger, Hart, Hirsch, Lynch, & Friedlaender, 2008). These mechanisms enhance the function of cells responsible for regeneration, including osteoblasts, cementoblasts, and fibroblasts (Bashutski & Wang, 2011; Hollinger et al., 2008; Sarment et al., 2006). Recombinant human PDGF‐BB contains 1,000 times more active growth factor than either PRP or platelet‐rich fibrin (Badylak et al., 2017; Nevins et al., 2005). Studies have shown that rhPDGF‐BB can stimulate bone fill and the rate of clinical attachment in periodontal defects (Nevins et al., 2005), and PRP can enhance soft tissue healing during the immediate postoperative period (Albanese et al., 2013).

The use of biologic agents is relatively new in regenerative dental applications. Evidence is still limited regarding their benefit in increasing the amount of bone formation in guided bone regeneration procedures when compared to a bone graft alone and whether they critically enhance the outcomes of ridge augmentation and ultimately improve success in implant placement (Bashutski & Wang, 2011; Del Amo et al., 2015; Dohan et al., 2006; Eskan et al., 2014).

For assessment of alveolar bone dimensions and anatomical structures prior to implant placement, three‐dimensional radiographs have become the standard (Deeb et al., 2017). Cone‐beam computed tomography (CBCT) provides accurate information about the alveolar ridge, and can be used for evaluation of both preoperative and postoperative alveolar ridge dimensions (Aimetti et al., 2018).

The aim of this retrospective study was to evaluate if the addition of biologic agents (PRP and rhPDGF‐BB) to a particulate bone graft results in a greater amount of horizontal ridge augmentation and greater augmented bone density when assessed radiographically, as well as if it improves the ability to place the implant.

## 2 MATERIALS AND METHODS

### 2.1 Study design

A retrospective chart review of implant patients from the Graduate Periodontics and the Oral and Maxillofacial Surgery clinics who had undergone horizontal ridge augmentation between 2013 and 2017 was completed. The study was approved by the Institutional Review Board at Virginia Commonwealth University (IRB approval HM20004398), and was conducted in accordance with the Helsinki Declaration of 1975, as revised in 2013.

A total of 188 horizontal ridge augmentation sites were initially identified through the chart review. Exclusion criteria involved having simultaneous horizontal ridge augmentation and implant placement, use of graft materials other than particulate bone grafts, use of non‐resorbable membranes, augmentations involving techniques other than tenting screws, and those patients without a preoperative and postoperative CBCT scan. Edentulous patients were excluded because of the need of dentition as reference points for the radiographic measurements. After these exclusions, the sample size was narrowed to 52 augmented sites. Twenty‐one of the 52 sites were treated with a particulate bone graft and adjunctive biologic agents (rhPDGF‐BB [*n* = 7] or PRP [*n* = 14]), while 31 received only a particulate bone graft.

## 3 SURGICAL TECHNIQUE

Ridge augmentation procedures were performed via a crestal incision over the edentulous ridge and full‐thickness mucoperiosteal flaps on the buccal and lingual aspects. Vertical releasing incisions and extensive split‐thickness dissections were performed to ensure tension‐free closure over bone graft and membrane. Deficient sites received a mixture of various proportions of particulate mineralized freeze‐dried bone allograft^1^ ^,^ ^2^ ^,^ ^3^ and bovine‐derived xenograft.^4^ Biologic agents (rhPDGF‐BB or PRP) were mixed into the particulate bone graft in 21 cases. The bone graft was supported by 6 mm long tenting screws,^5^ of which 3 mm served for intraosseous retention and 3 mm to support the particulate bone graft (Le & Rohrer, 2017). The bone graft was covered with a resorbable collagen membrane^6^ ^,^ ^7^ ^,^ ^8^ or an acellular dermal matrix for guided bone regeneration.^9^ ^,^ ^10^ The barriers were secured with tacks or resorbable subperiosteal sutures^11^ (Urban, Lozada, Wessing, Suárez‐López del Amo, & Wang, 2016). All patients received a preoperative oral dose of Amoxicillin 2 g or Clindamycin 600 mg, followed by 500 mg Amoxicillin or 300 mg Clindamycin TID for 1 week, and analgesics, as needed, to manage postoperative discomfort. Patients were advised to use 0.12% Chlorhexidine mouth rinse twice daily for 2 weeks following the surgery. Postoperative visits were scheduled at 1, 4, and 24 weeks with postoperative CBCT scheduled at 24 weeks.

## 4 RADIOGRAPHIC ASSESSMENT

For assessment of gains in alveolar ridge bone width, the cross‐sections of the grafted area were measured on the preoperative CBCT scans and compared to images taken, on average, 7.3 months postoperatively (Table 1). Since this was a retrospective chart review, there was not a standardized time at which the final postoperative CBCT was taken for each patient.

**TABLE 1:** Sample demographics and baseline measures

Sample demographics						
	Overall		Biologics (*n* = 21, 40%)		Non‐biologics (*n* = 31, 60%)	
	*n*	%	*n*	%	*n*	%
Gender						
Male	13	25	7	33	6	19
Female	39	75	14	67	25	81
Jaw						
Maxilla	17	33	9	43	8	26
Mandible	35	67	12	57	23	74
Position in mouth						
Anterior	10	19	9	43	1	3
Posterior	42	81	12	57	30	97
	Mean	*SD*	Mean	*SD*	Mean	*SD*
Age	60.96	11.73	57.71	14.74	63.16	8.76

Baseline measures						
	Overall		Biologics (*n* = 21, 40%)		Non‐biologics (*n* = 31, 60%)	
	Mean	*SD*	Mean	*SD*	Mean	*SD*
Baseline bone level (width) (mm)	5.39	1.32	5.06	0.99	5.62	1.48
Follow‐up bone level (width) (mm)	8.88	2.03	8.64	2.09	9.03	2.00
Average augmentation (mm)	3.59	1.84	3.58	1.96	3.60	1.79
Postop bone graft density	1,025.15	515.94	1,130.81	384.80	953.58	583.70
Time between grafting and postop CBCT (months)	7.33	4.19	6.33	2.27	8.00	5.03

**FIGURE 1:**
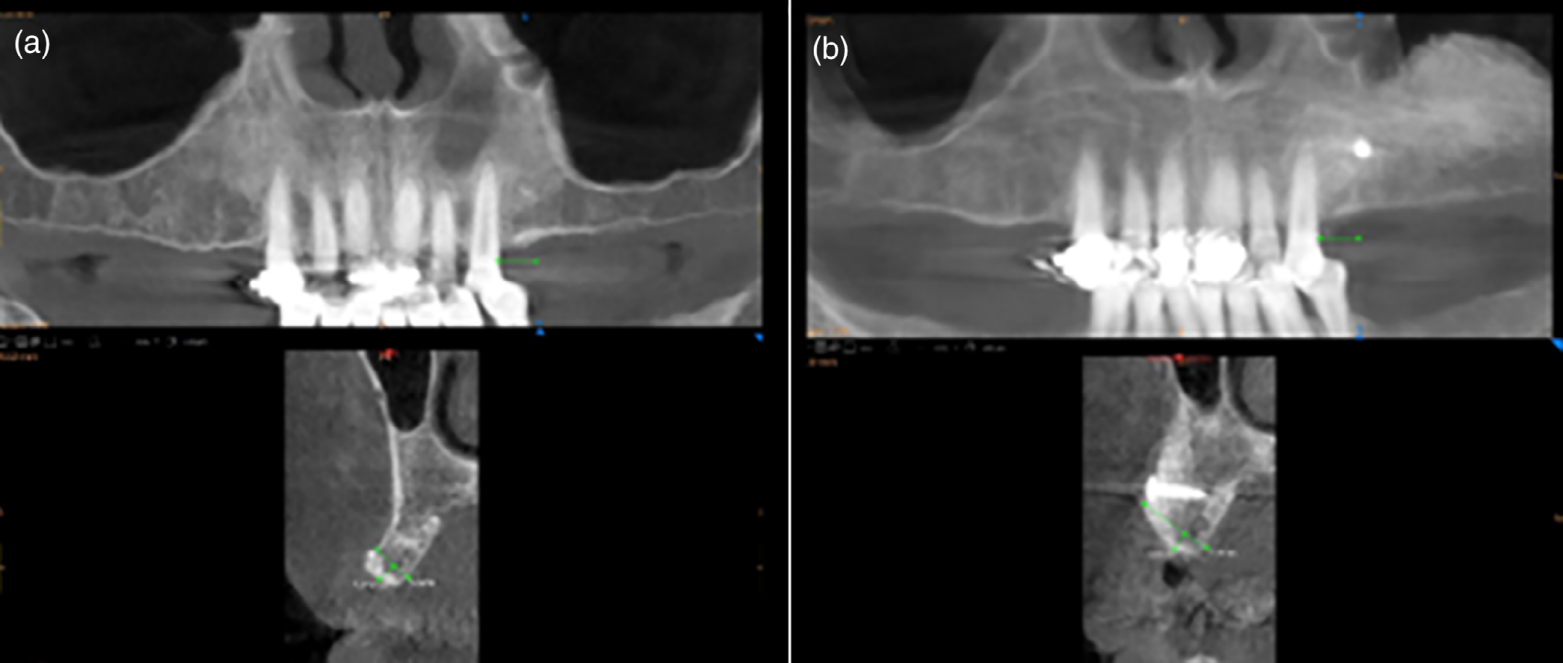
Radiographic analysis with linear measurement of alveolar ridge before (a) and after (b) augmentation

CareStream CBCT scan software^12^ was used for the horizontal measurements, and bone density readings were based on the grayscale (Figure 1). The gray levels in the standard CT images were then converted into a quantitative scale of specific Hounsfield units (HU) (Mah, Reeves, & McDavid, 2010). Two independent raters measured a subset of the cases to test for accuracy of the measurements. The intraclass correlation coefficient (ICC) of the two raters was high, at 0.94. Therefore, one rater then completed the rest of the measurements used for the analysis. The horizontal dimensions of the alveolar ridge in the desired implant sites were measured before and after augmentation of 3 mm apical to the alveolar crest at three set points 3 mm apart. The distal surface of the nearest adjacent tooth served as a reproducible reference point for the measurements. The three horizontal measurements were averaged to create a single value. These values were analyzed statistically to study bone augmentation outcomes and compare sites treated with and without biologics.

## 5 STATISTICAL METHODS

Patient demographics were summarized using descriptive statistics. The effect of the biologics on the ridge augmentation outcomes and density of the grafted bone were first tested using ANCOVA, adjusting only for the time since surgery and use of biologics. Overall models for postoperative bone level were then adjusted for age, gender, jaw (maxilla, mandible), and location (posterior, anterior). Backwards elimination was used to find a parsimonious model; however, variables of interest (use of biologics and time since surgery) were maintained in all the models.

## 6 RESULTS

**FIGURE 2:**
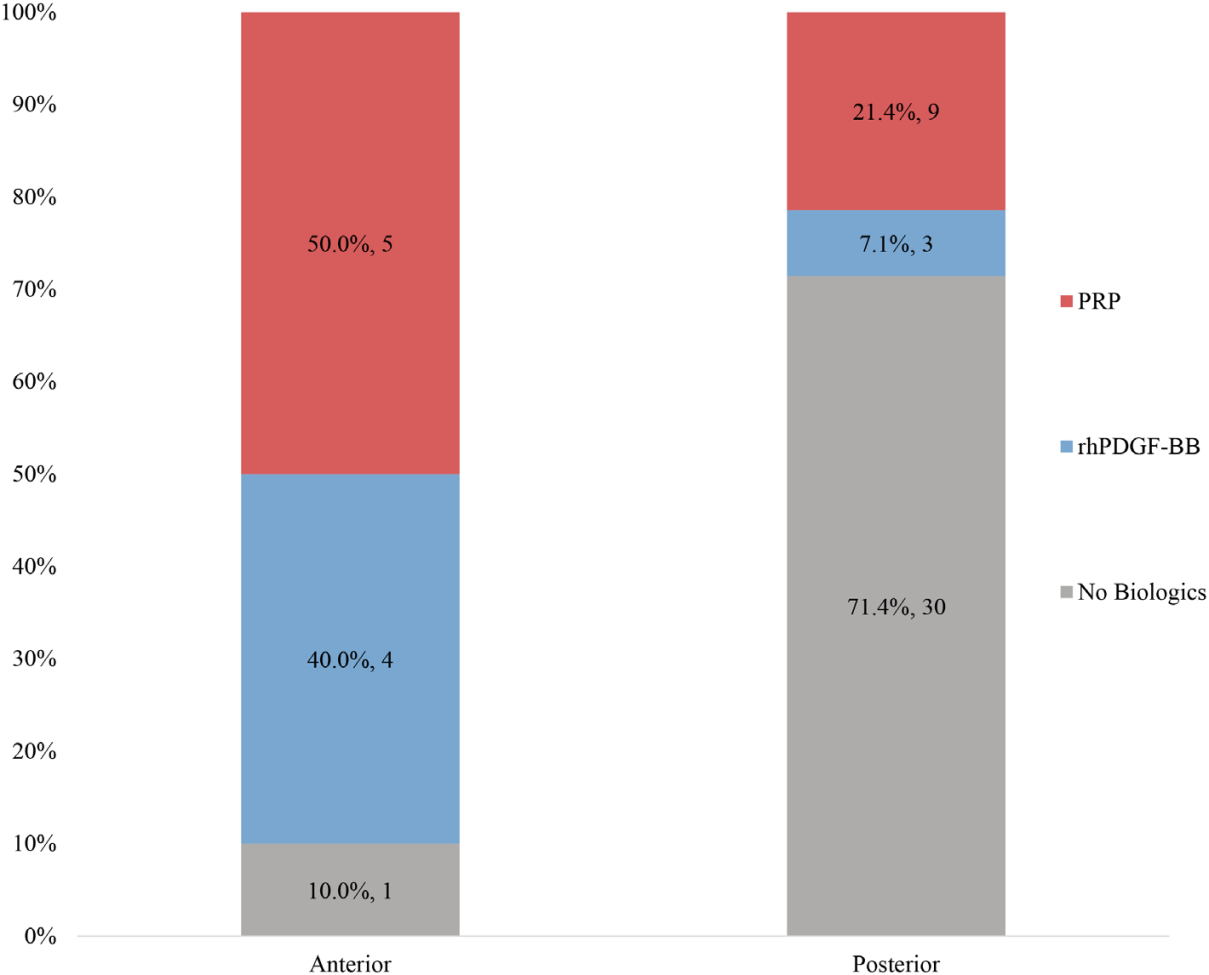
Comparison of frequency and type of biologics used for anterior versus posterior sites

A total of 43 patients and 52 augmented sites were included in the analysis. The number of implant sites ranged from 1 to 4 with an average of 1.2 sites per patient. Patient demographics are given in Table 1. They ranged in age between 31 and 79 years (average of 60.8 years; *SD* = 12.1). Most patients were female (*n* = 31, 72%). Almost half of the patients received biologics (*n* = 21, 40%). Of those, 14 received PRP and 7 received rhPDGF‐BB. Patients who received biologic agents were similar to those who did not in terms of age (*p* value = .1388), gender (*p* value = .3325), jaw (*p* value = .2378), and baseline bone level (*p* value = .1157), but the follow‐up time between the ridge augmentation date and the postoperative CBCT scan was marginally shorter for those who received the biologic agents (6.3 months vs. 8.0 months, *p* value = .1128). Significantly more anterior sites than the posterior areas of the mouth were treated with biologic agents (90.0 vs. 28.5%, *p* value = .0006) (Figure 2). Among the cases that received biologics, the selection of rhPDGF versus PRP was not associated with implant site (rhPDGF‐BB: 57% anterior vs. 43% posterior; *p* value = .3972). Augmented sites were able to receive implants in 98% of the cases (*n* = 51). One patient did not return for implant placement and the outcome is unknown.

### 6.1 Bone augmentation

**FIGURE 3:**
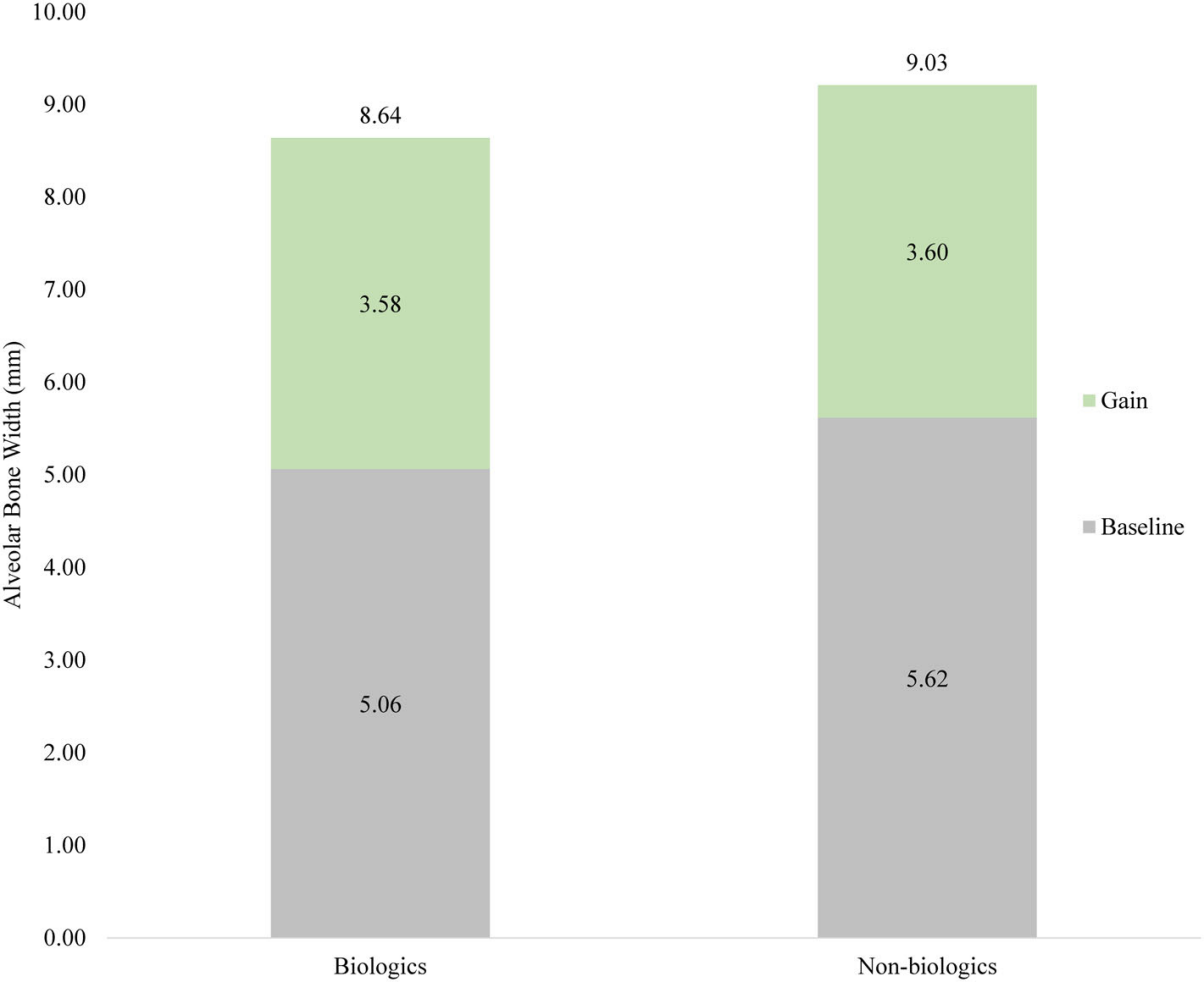
Average baseline alveolar ridge width, bone gain, and postoperative alveolar ridge width for biologics and non‐biologics ridge augmentation groups (in mm)

Table 2 summarizes the baseline and postoperative alveolar ridge width findings. The average initial width of the alveolar ridge in the two groups of patients averaged 5.39 mm, with an average of 5.62 in the non‐biologic group and 5.06 in the biologics group (*p* value = .1411). The average horizontal bone gain for all cases combined was 3.6 mm (*SD* = 1.84), which was a statistically significant gain (*p* value < .0001; 95% CI: 3.04–4.07). The horizontal gain by group was nearly identical with 3.58 mm (*SD* = 1.96) for the biologics group and 3.60 for the non‐biologics group (*SD* = 1.79). The postoperative width in the non‐biologic group was 9.09 mm versus 8.73 mm in the biologic group (Figure 3). This difference was not statistically significant (Table 2; *p* value = .5094).

**TABLE 2:** Initial association between bone level, bone density, and use of biologics

	Mean^a^	95% CI	*p* value
Postoperative bone level (width)			
Biologics	8.73	7.93 to 9.54	
No biologics	9.09	8.42 to 9.76	
Difference	−0.35	−0.72 to 1.43	.5094
Bone graft density			
Biologics	1,143.87	757.08 to 1,132.39	
No biologics	944.73	915.01 to 1,372.74	
Difference	199.14	−99.62 to 497.90	.1866

a Mean adjusted for time since bone graft and baseline bone level (for postoperative bone level).

The overall model for change in alveolar bone width considered the following factors: baseline bone width, healing time, use of biologics, patient age and gender, jaw (maxilla, mandible), and location in the mouth (posterior, anterior). Factors that were not statistically significant were removed with backwards elimination in the following order: jaw (*p* value = .6117), gender (*p* value = .5324), age (*p* value = .3829). The final model based on biologics included the baseline bone width (*p* value = .0294), bone width at 1–24 months after surgery (*p* value = .0961), and grafting site location in the mouth (posterior, anterior) (*p* value = .0172) (Figure 2). When adjusting for all these factors, the use of biologic agents still did not significantly increase postoperative bone augmentation (*p* value = .6196). However, greater baseline bone width was associated with a greater increase in postoperative bone width (Table 3). For each 1 mm of increase in baseline bone width, there was an associated postoperative gain of 0.45 mm. Posterior sites had greater postoperative bone width than anterior sites (9.31 vs. 7.54 mm) (Table 3).

**TABLE 3:** Adjusted model for association between bone level and biologics

	Estimated mean postoperative bone level	95% CI	*p* value
Baseline bone level (width) (1 mm increase)	0.45	0.05 to 0.84	.0294
Biologics (Y/N)			.6196
Yes	8.57	7.79 to 9.35	
No	8.29	7.37 to 9.20	
Months from surgery (1 month increase)	−0.10	−0.22 to 0.02	.0961
Location			.0172
Anterior	7.54	6.33 to 8.76	
Posterior	9.31	8.73 to 9.90	

### 6.2 Bone graft density

The initial model for evaluating postoperative bone density included only the healing time (defined by the time between the date of surgery and the postoperative CBCT) and the use of biologics. The use of biologics was not associated with a significant difference in bone density (Table 2; *p* value = .1866).

The overall model for bone graft density considered the following factors: healing time, use of biologics, patient age and gender, jaw (maxilla, mandible), and location in mouth (posterior, anterior). Factors that were not statistically significant were removed with backward elimination in the following order: location in the mouth (*p* value = .5678), jaw (*p* value = .6165), and age (*p* value = .5439). Patient gender was retained in the final model due to marginal statistical significance (*p* value = .0944). Healing time (*p* value = .1582) and use of biologics (*p* value = .0841) were also retained as design variables in the study. The estimated bone graft density for patients who received biologics was 1,106.19 HU (*SE* = 113.89) compared with 839.64 HU (*SE* = 110.39) for those who did not (average difference: 266.55, 95% CI: −37.27, 570.38). Mean bone density was higher for sites treated with PRP (1,184.36) than the sites treated with rhPDGF‐BB (1,023.71). Females had a marginally significantly greater postoperative bone density than males (1,128.65 vs. 817.17, 95% CI on difference: −55.57, 678.54). The results are summarized in Table 4.

**TABLE 4:** Adjusted model for association between bone graft density and use of biologics

	Estimated bone density	95% CI	*p* value
Biologics (Y/N)			.0841
Yes	1,106.19	877.19 to 1,335.18	
No	839.64	617.68 to 1,061.60	
Months from surgery (1 month increase)	27.47	−11.06 to 66.00	.1582
Gender			.0944
Male	817.17	513.30 to 1,121.04	
Female	1,128.65	953.79 to 1,303.52	

## 7 DISCUSSION

Alveolar ridge deficiencies often require surgical correction prior to implant placement. In contemporary practice, resorbable collagen membranes and particulate bone allografts are frequently used for alveolar ridge augmentation despite limited published evidence to support their clinical application (Elangovan, Barwacz, Antonious, Swenson, & Avila‐Ortiz, 2017). Several studies have shown adequate alveolar bone augmentation outcomes with the use of bone grafts and membranes (Aghaloo & Moy, 2007; Beitlitum et al., 2010; Esposito, Grusovin, Kwan, Worthington, & Coulthard, 2009; Esposito et al., 2009; Lekovic et al., 1997; McAllister & Haghighat, 2007; Wang & Boyapati, 2006). The guidelines for the use of biologic agents in horizontal ridge augmentation are still evolving. Such agents have been successfully applied in the repair of osseous defects (Darby & Morris, 2013). A recent literature review demonstrated that regenerating horizontal ridge defects can be enhanced by rhPDGF‐BB in in vitro and animal preclinical studies; however, its specific contribution and effectiveness in human alveolar ridge augmentation is inconclusive (Scheines, Hokett, & Katancik, 2018). The evidence based on animal biopsies suggests that the addition of biologics may increase the number of marrow cells but it does not have a significant effect on bone regeneration (Yoon et al., 2014). The outcomes of the clinical studies suggest enhanced healing and faster bone graft turnover in the presence of rhPDGF‐BB. However, the addition of growth signaling molecules does not have a statistically significant effect on either vital bone gain or bone density when compared to a bone graft alone (Wallace, Snyder, & Prasad, 2013).

Clinicians may be more likely to add biologic agents in cases of greater severity where obtaining significant augmentation is more challenging. The same is true for esthetic areas with high visibility and patient expectations. The addition of biologic agents in this study occurred more frequently for ridge augmentation of anterior areas, likely with the aim of optimizing the outcome in the esthetic zone with its more challenging anatomy. This may cause some bias in the results since the use of biologics was not randomized equally to anterior and posterior sites. A randomized controlled trial would be necessary to fully mitigate this potential confounding factor.

Radiographic evaluation of all 52 sites, which involved the use of tenting screws, showed an average horizontal bone gain of 3.6 mm, which is similar to data from other ridge augmentation studies (Caldwell, Mills, Finlayson, & Mealey, 2015). Studies using resorbable membranes without tenting screws reported horizontal gains of only 1.65 mm (Sterio, Katanick, Blanchard, Xenoudi, & Mealey, 2013). Although the use of PRP or rhPDGF‐BB was not associated with significant improvements in the amount of horizontal bone gain, enhanced bone density was observed. However, the difference was only marginally statistically significant. At reentry surgery, both types of augmented sites appeared to have similar clinical characteristics and were suitable for placement of the desired number of implants of preferred dimensions in the planned positions (Figure 3).

**FIGURE 4:**
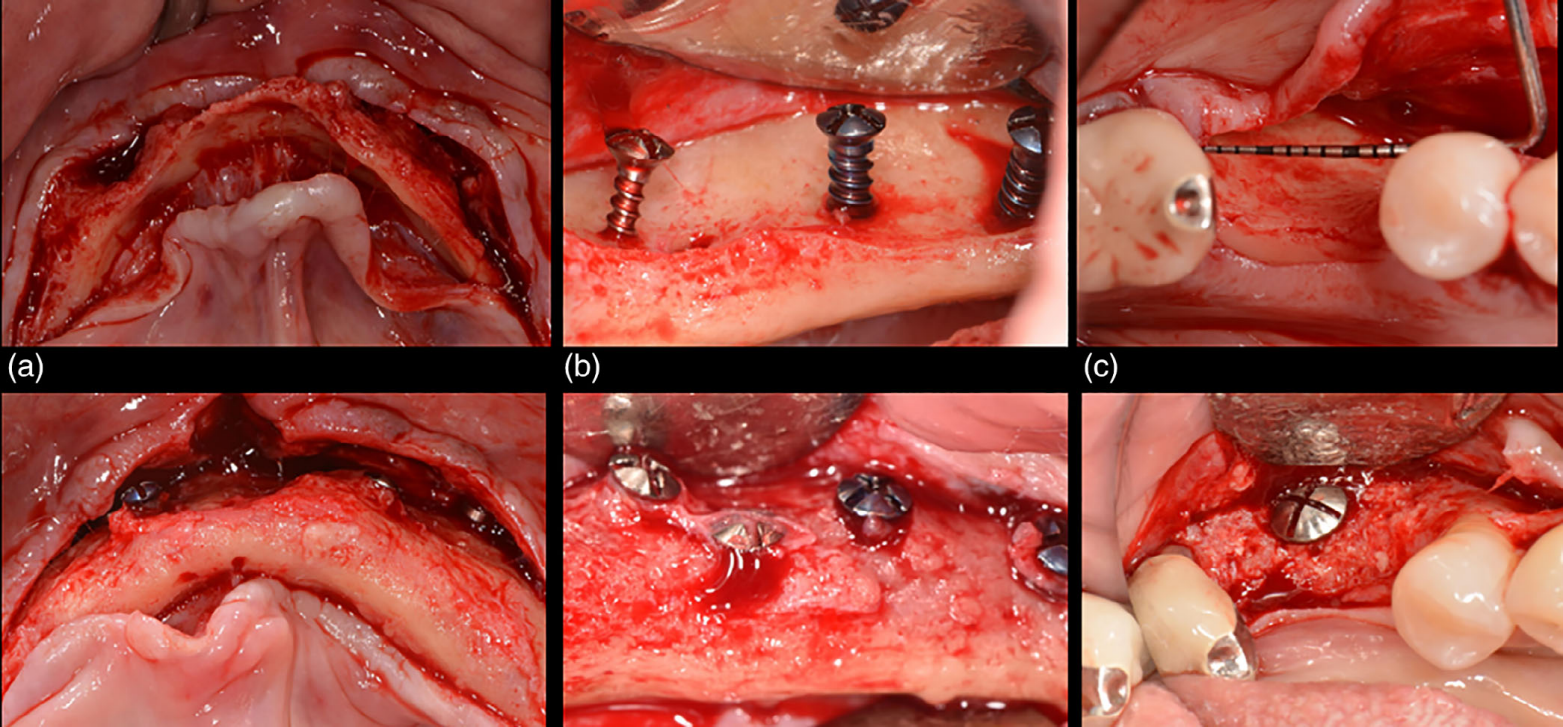
Clinical view of alveolar ridge augmentation using tenting screws and bone graft and platelet‐rich plasma (PRP) (a), bone graft with no biologics (b), and bone graft and recombinant human platelet‐derived growth factor‐ββ (rhPDGF‐BB) (c); before (first row images) and after augmentation (second row images)

In clinical cases, rhPDGF‐BB has been credited with increased vital bone formation and accelerated remodeling of allografts and xenografts (Wallace, Snyder, & Prasad, 2013), and with significant improvement in clinical attachment levels and bone fill in the treatment of periodontal defects (Nevins et al., 2005), which has enhanced effect on periodontal ligament cell proliferation when combined with a particulate bone allograft (Papadopoulos et al., 2003; Figure 4).

The use of PRP has consistently shown improvement in soft tissue healing, but its benefits in bone regeneration are controversial (Albanese et al., 2013; Alissa, Esposito, Horner, & Oliver, 2010). PRP may enhance bone regeneration (Eskan et al., 2014), and the grafted sites in this study showed increased bone density with both PRP and rhPDGF‐BB. Although the addition of biologic agents marginally improved the mineral bone density, significant conclusions cannot be drawn from the results due to the wide confidence interval implying a great amount of variability resulting from the relatively small sample size.

This study has several limitations. The patient population presented a wide range in age, which could affect new bone formation. A variety of brands of resorbable membranes and particulate bone graft materials were used, possibly introducing slight differences that could affect the outcomes. Timelines for the radiographic bone measurements also differed among patients, introducing variability. Some CBCT scans were taken early due to postoperative complications (infection, swelling, neurosensory disturbances, and loose hardware) or late due to patients scheduling preferences, thus widening the range. The fact that teeth were needed as reference points for radiographic measurements, which eliminated all edentulous patients from the study.

Linear measurements of the augmented bone were performed rather than a volumetric analysis based on the available software. Although linear radiographic measurements provide valuable information regarding alveolar bone width gain, and have been used in similar studies (Hong, Chen, Kim, & Machtei, 2019), these measurements provide only a two‐dimensional assessment compared to a volumetric analysis achieved by superimposition of three‐dimensional images (Koerich, Weissheimer, Koerich, Luz, & Deeb, 2018).

To provide better guidance for the application of biologic agents in ridge augmentation procedures, evidence to support their use should be consistent with the specific procedure. To further investigate the ability of biologic agents to improve the outcomes of guided bone regeneration procedures, a prospective study with better controlled variables is indicated.

## 8 CONCLUSION

The use of the tenting screws and resorbable membranes in combination with a mixture of particulate bone allograft and xenograft provides significant dimensional changes in alveolar ridge width for the placement of implants of the appropriate size in the desired location. Within the limitations of this retrospective study, the addition of biologic agents to the graft materials resulted in marginally improved bone density but did not make a significant difference in the amount of bone gain or the ability to place implants.

## CONFLICT OF INTEREST

The authors declare no conflicts of interest.

## Endnotes

^1^
Endobond, Zimmer Biomet, Warsaw, IN.

^2^
Exactech Optecure.

^3^
Dynablast, Keystone Dental, Burlington, MA.

^4^
Bio‐Oss, Geistlich Pharma North America, Princeton, NJ.

^5^
Salvin Dental Specialties, Charlotte, NC.

^6^
Bio‐Gide, Geistlich Pharma North America, Princeton, NJ.

^7^
Dynamatrix, Keystone Dental, Burlington, MA.

^8^
Optimatrix, Osteohealth, Shirley, NY.

^9^
Alloderm GBR, Biohorizons, Birmingham, AL.

^10^
Meisinger, Neuss, Germany.

^11^
Chromic gut, Ethicon, Somerville, NJ.

^12^
CS 8100 3D, Carestream Dental, Rochester, NY.
